# Good news or bad news? The coronavirus pandemic has sickened and killed only a relatively few people but has affected us all

**DOI:** 10.3325/cmj.2020.61.296

**Published:** 2020-06

**Authors:** Charles H. Calisher

In 1846, the Danish Government sent Peter Ludwug Panum to the Faroe Islands to investigate an epidemic. This physiologist-pathologist completed an excellent epidemiological investigation and made many fundamental observations, including that infections of people with whatever was causing the disease (ie, measles virus) led to development of life-long resistance (ie, immunity) to it. From that period until now, we have been informed and comforted by the knowledge gained from Panum’s studies (1). The current pandemic of the newly recognized severe acute respiratory syndrome betacoronavirus-2 (SARS-CoV-2) may very well change or modify our general understandings. Given that the world’s human population is about 7.8 billion, as of May 22, 2020 only ~ 0.07% of the population has been shown to have been infected with this virus worldwide ( ~ 0.48% in the U.S.A.). Unfortunately, ~ 6.5% of those shown to be infected ( ~ 6% in the U.S.A.) have died. These are frightfully large and wretched numbers, reflecting an enormous amount of suffering and sadness, not to speak of monetary cost, extra work and extra risk for first responders and hospital workers, the need for new tools, political unrest, unemployment, personal distancing, marital discord, school closings, food shortages, severe stock market decreases, inconveniences (the least problem), and the shockingly high rate of incapacitation and deaths among health care workers and other first responders; and these bare infection rate data do not take into account the huge number of people who have not been tested for the virus. About 3.4% of symptomatic individuals require hospitalization (7.4% of those 65 years of age or older), about 40% of transmission occurs from asymptomatic people, and 0.4% of symptomatic patients die (1.3% of symptomatic patients 65 years of age or older).

Furthermore, note must be made of the “essential workers,” those whose services keep the rest of us supplied with food, clean hospitals, care of the elderly, etc, but whose work pays so little that they cannot afford or choose to “stay at home.”

## The present situation

As of the time of this writing, world-wide there have been more than 5 million cases of infection with the coronavirus SARS-CoV-2 and more than 335 000 of these patients have died. The first recognized human infection occurred in China no later than December 1, 2019 and the originally recognized outbreak was reported to the World Health Organization on December 31, 2019 (2). Note: I presume that cases are defined by illnesses (a 2- to 14-day incubation period followed by at least fever, dry cough, shortness of breath or difficulty breathing, headache, sore throat, repeated shaking with chills, fatigue, muscle (especially chest) pain, and recent loss of taste or smell) confirmed by laboratory testing (polymerase chain reaction, I also presume). The numbers of these diagnosed cases surely are much lower than the actual number of infections (people who have become infected with SARS-CoV-2, whether ill or not). Millions of people worldwide have not been tested at all. In the U.S.A. alone, there have been more than one and a half million diagnosed cases, with well more than 95 000 deaths. In addition, cardiac arrest in apparently asymptomatic pregnant women has been observed, and certain patients of many age-related cohorts have manifested remarkable peculiarities including: lack of fever, clotting aberrations (“hemostatic derangements”) causing strokes and sudden death, appendage rashes/reddening (“COVID toes,” “Kawasaki rash”), Guillain-Barré syndrome, and other complications. Indeed, hemostatic disorders have been observed in up to 40% of patients with this virus and may be responsible for the deaths of many or most of them. Immunologic imbalances, for example interleukin-6-mediated depletion of certain important lymphocytes with sustained cytokine production and resulting hyperinflammation, or other mechanisms, can bring about cytokine storms, such as those making the 1918 influenza virus pandemic so pathogenic for older or very young patients.

## What might be next?

That is the key question. Should everyone not showing evidence of infection with SARS-CoV-2 be released from voluntary and involuntary personal distancing in order to restart national economies? Those many millions who have not been infected, and who therefore remain susceptible to this virus, might serve as hosts for the virus, and the apparent control brought about through the hard work and contributions of millions of people would almost assuredly be lost. That would result in an even more extensive pandemic than we currently have. The problem with these numbers is that at this time we have no idea how many asymptomatic infections there have been. Current estimates suggest that as many as 25% of infected individuals show no signs of illness. In light of this, we do not know whether the longer the pandemic persists, the closer we approach “herd immunity” and, therefore, the closer we would be to a reduced likelihood of a susceptible individual contacting an infected individual. All this and more are dependent upon the immunological responses of humans to SARS-CoV-2 and other coronaviruses.

## Many research questions remain to be fully investigated

What is the natural host of SARS-CoV-2? Coronaviruses have been detected in pangolins, also known as scaly anteaters (placental mammals of the order Pholidota, family Maninae, genus *Manis*) and in bats of many species; bats also are placental mammals but of the order Chiroptera). At present, it is hypothesized that SARS-CoV-2 may have originated in Asian horseshoe bats (family Rhinolophidae, including *Rhinolophus affinis*) (2). It then somehow adapted to pangolins or to other live vertebrates, perhaps, but not proven to have been, in “wet markets” (now outlawed in China), then transmitted to humans. Unless we determine the natural hosts of this virus, including determining other potential natural hosts in regions where the virus is not now known to occur, whether it can be transmitted by arthropods (unlikely, but a nightmare to contemplate), and whether SARS-CoV-2 can be transmitted from humans to other vertebrates (“spillback”), such as bats, we may be co-existing with this virus for the foreseeable future (3). Finding that wild vertebrates, including rodents, are transmission competent hosts would be devastating with regard to establishment of endemicity.

The current pandemic has been tentatively associated with the aforementioned “wet” market in Wuhan, China, where wild animals may have been the source of this zoonotic. Although bats are likely reservoir hosts for SARS-CoV-2, the identity of an intermediate host that might have facilitated transfer to humans is unknown. Lam et al (4) have reported the identification of a SARS-CoV-2-related coronaviruses in Malayan pangolins (*Manis javanica*) seized in anti-smuggling operations in southern China. Metagenomic sequencing identified pangolin-associated coronaviruses that belong to two sub-lineages of SARS-CoV-2-related coronaviruses, including one that exhibits strong similarity to SARS-CoV-2 in the receptor-binding domain. The discovery of multiple lineages of pangolin coronavirus and their similarity to SARS-CoV-2 suggests that pangolins should be considered as possible hosts in the emergence of novel coronaviruses and should be removed from all markets to prevent zoonotic transmission (4,5).

It may be that bat immune defenses may drive the evolution of viruses that are rapidly transmitted, and while bats are well protected from the harmful effects of their own prolific viruses, other creatures, such as humans, are not. Such findings may help to explain why bats are often the source for viruses that are deadly in humans. Learning more about the antiviral defenses of bats and how they drive virus evolution may help scientists develop better ways to predict, prevent, or limit the spread of viruses from bats to humans. More bat studies are needed to support these efforts. In the meantime, these experiments highlight the importance of warning people to avoid direct contact with wild bats.

Some additional questions:

Do IgA, IgM, IgG, IgD, IgE, and perhaps other immunoglobulins produced against infection with the virus actually neutralize the virus (protect against it)?

Do antibodies to the virus persist? As antibody titer decreases with time could viral recrudescence occur and periodic outbreaks or epidemics follow?

Is reinfection of humans possible?

Is “spillback” (from humans or other vertebrates to bats) possible and significant?

Do the peculiarities of this particular virus bring about antibody-dependent enhancement, making patient survival or uncomplicated recovery less likely?

Will a vaccine against SARS-CoV-2 complicate or even counteract its intent?

Might latent infections in newborn or young children lead to virus persistence through life, as with varicella-zoster virus?

Are there racial and gender differences in susceptibility? Do X chromosomes influence susceptibility and severity of illness caused by SARS-CoV-2?

What are the host and virus characteristics of infections leading to partial protection (with virus shedding)?

For how long does a SARS-CoV-2-infected human shed virus, from pre-symptomatic to post-symptomatic (if recovered)? For how long does an asymptomatic human shed virus? How much virus?

Is SARS-CoV-2 shed only from mucous membranes? How about from feces?

Can the virus be sexually transmitted?

Should re-examination of poorly diagnosed illnesses be done to rule out a SARS-CoV-2 causation?

Why are older patients and particularly those with co-morbidities more prone to die with this infection, as has been shown to occur with Middle East respiratory coronavirus?

Of 175 Chinese patients with mild symptoms of SARS-CoV-2, 70% developed strong antibody responses, but about 25% had poor responses and about 5% developed no detectable antibody. It could be that mild illness might not always result in protection. It will be important to study the immune responses of people with asymptomatic SARS-CoV-2 infection to determine whether symptoms, and their severity, predict whether a person becomes immune (6). Can “silent spreaders” (asymptomatic, pre-symptomatic, and very mildly symptomatic) be identified and their infections quenched? For how long do these people spread the virus and with what intensity?

**Figure 1:**
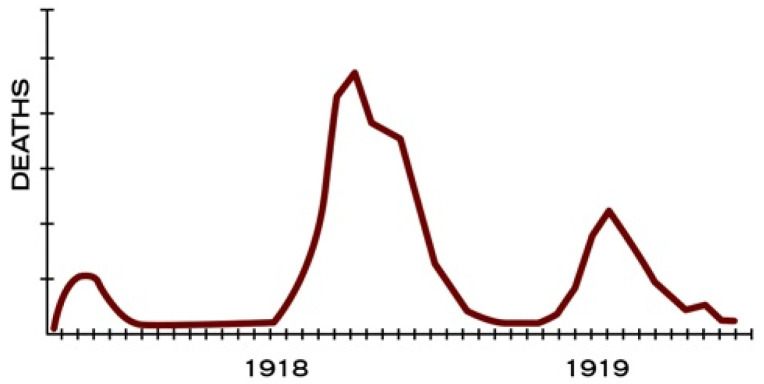
1918 influenzavirus pandemic deaths by year, 1918-1919. Source: *https://www.cdc.gov/flu/pandemic-resources/1918-commemoration/three-waves.htm*.

During the mis-named 1918-1919 “Spanish flu” pandemic, there were three different waves of illness, starting in March 1918 and subsiding by summer of 1919 (Figure 1). The pandemic peaked in the US during the second wave, in the fall of 1918. This highly fatal second wave was responsible for most of the US deaths attributed to the pandemic. Will the current coronavirus pandemic be similar to the 1918 influenza pandemic? Is it inevitable that next year or a year after that the world again will go through the present experience, with great loss of life and economic catastrophe? Will this occur repeatedly in the future? Or will the current pandemic simply persist until an adequate vaccine or other preventative becomes available?
